# Human Placental Extract Delays In Vitro Cellular Senescence through the Activation of NRF2-Mediated Antioxidant Pathway

**DOI:** 10.3390/antiox11081545

**Published:** 2022-08-10

**Authors:** Liguo Huang, Lit-Chein Chin, Koichi Kimura, Yasukazu Nakahata

**Affiliations:** 1 Department of Research & Development, Melsmon Pharmaceutical Co., Ltd., Tokyo 171-0014, Japan; 2 Department of Neurobiology and Behavior, Graduate School of Biomedical Sciences, Nagasaki University, Nagasaki 852-8523, Japan

**Keywords:** cellular senescence, human placental extract, oxidative stress, dermal fibroblasts

## Abstract

Senescent cells accumulate in the organs of aged animals and exacerbate organ dysfunction, resulting in age-related diseases. Oxidative stress accelerates cellular senescence. Placental extract, used in the alleviation of menopausal symptoms and promotion of wound healing and liver regeneration, reportedly protects against oxidative stress. In this study, we investigated the effects of human placental extract (HPE) on cellular senescence in normal human dermal fibroblasts (NHDFs) under oxidative stress conditions. We demonstrated that HPE delays the onset of cellular senescence. Next-generation sequencing analysis revealed that under oxidative stress conditions, HPE treatment enhanced the expression of the antioxidant genes *CYGB*, *APOE*, *NQO1*, and *PTGS1*. Further, HPE treatment under oxidative stress conditions increased the protein level of nuclear factor-erythroid factor 2-related factor 2 (NRF2)—a vital molecule in the antioxidant pathway—via post-transcriptional and/or post-translational regulations. These findings indicate that HPE treatment in NHDFs, under chronic oxidative stress, delays cellular senescence by mitigating oxidative stress via upregulation of the NRF2-mediated antioxidant pathway, and HPE treatment could potentially ameliorate skin-aging-associated damage, in vivo.

## 1. Introduction

Skin aging has become one of the most common dermatological concerns of the aging population globally. It is triggered by both intrinsic and extrinsic factors [1]. Skin aging occurs naturally during chronological aging. External environmental factors, such as ultraviolet radiation (UVR), environmental pollutants, smoking, and microbial insults can accelerate skin aging and lead to premature skin aging [2,3,4,5]. Among these, UVR is considered to be the dominant factor in premature skin aging. Although UVC (100–290 nm) is largely absorbed by the ozone layer, UVB (290–320 nm) and UVA (320–400 nm) penetrate the epidermis and dermis, respectively [2]. UVA radiation induces oxidative stress in keratinocytes and fibroblasts in the dermis, by stimulating the production of reactive oxygen species (ROS) and other free radicals. UVR is responsible for DNA damage [1], and chronic exposure to UVR leads to skin diseases, such as skin cancer [6]. Furthermore, oxidative stress can accelerate premature cellular senescence. Accumulation of senescent cells in tissues (including cutaneous tissue) of aged animals, exacerbates tissue dysfunctions and results in age-related diseases. Therefore, chronic exposure to UVR is the most harmful environmental factor that leads to premature skin aging, also known as “photoaging”.

The nuclear factor erythroid factor-2-related factor 2/Kelch-like ECH-associated protein 1 (NRF2/KEAP1) pathway is the major regulator of cytoprotective responses to oxidative stress [7,8,9]. NRF2, an inducible transcription factor activated by oxidative stress, activates a wide array of genes, encoding antioxidant proteins. This activity is tightly regulated by KEAP1, which promotes the degradation of NRF2 via the ubiquitin-proteasome pathway. Reportedly, NRF2 activation not only protects cells from oxidative stress but also regulates cell proliferation, differentiation, and cancer chemoresistance [7,8,9].

Human placental extract (HPE) has been approved for clinical use [10,11,12,13,14]. It is now widely used to achieve skin whitening, improve health, and combat fatigue and aging [13,15,16]. Notably, HPE has been shown to mitigate oxidative stress at different levels: HPE ingredients, such as uracil, tyrosine, phenylalanine, tryptophan, and collagen-derived peptides, impair Fenton’s reaction [17,18]. HPE decreases ROS levels in cultured cells [19,20] and ameliorates tissue abnormalities induced by oxidative stress [20,21,22]. However, whether HPE slows oxidative stress-induced cellular senescence remains largely unknown.

In this study, we evaluated the mechanism by which HPE attenuates oxidative stress-induced cellular senescence in normal human dermal fibroblasts. Furthermore, the RNA sequencing and biochemical analyses suggested that HPE activates the master antioxidant pathway—the NRF2/KEAP1 pathway—in response to oxidative stress.

## 2. Materials and Methods

### 2.1. Materials

Human placental extract (HPE), obtained from Melsmon Pharmaceutical Co., Ltd., Tokyo, Japan, was used in this study [23]. The procedure to manufacture the HPE used in this study included the hydrolysis of human placenta from healthy donors, with acid, neutralized to pH 6.8–7.0, and sterilized at 121 °C for 30 min. The resulting HPE, containing a wide range of amino acids, nucleic acids, saccharides, and lipids [10,24], was adjusted to 50 mg/mL of human placental hydrolysate. H_2_O_2_ (3%) was purchased from Sigma-Aldrich, St. Louis, MU, USA.

### 2.2. Cell Culture

Normal human adult dermal fibroblasts (NHDFs) (Cat# 106-05a; Lot No. 2485; isolated from the normal human facial skin of a 66-year-old Caucasian woman), and cell culture ingredients, were purchased from Cell Applications, Inc., San Diego, CA, USA. The cells were cultured in an endothelial basal medium containing a 3% endothelial cell growth supplement at 37 °C with 5% CO_2_, in accordance with the manufacturer’s instructions. The cells used in this study were between passages 7 and 19, corresponding to individual population doubling levels (iPDL) of 4 and 0, respectively.

### 2.3. Cell Proliferation Assay

NHDFs were cultured in triplicate, in a 6-well plate for four days, trypsinized, counted, and seeded again in a 6-well plate until the cells ceased to proliferate. The cells were treated with: HPE; 50 μM H_2_O_2_; or HPE + 50 μM H_2_O_2_. Untreated cells were used as negative controls. The culture medium was replaced daily. We used the formula “*n* = 3.32 * log(Npost/Npre)” to calculate the PDL, where *n* = PDL number after 4-day culture, Npost = total cell number after 4-day culture, and Npre = seeding cell number. Cumulative PDL (cPDL) represents the accumulation of iPDL. The iPDL and cPDL were recorded by subculturing NHDFs every four days. In this study, we determined the onset of cellular senescence at *n* < 0.5.

### 2.4. Senescence-Associated β-Galactosidase Activity

Cells were rinsed with phosphate-buffered saline (PBS) and fixed with a 1x fixative solution, provided by a senescence-associated β-galactosidase staining kit (Cat#9860, Cell Signaling Technology, Danvers, MA, USA), for 15 min. A fresh β-galactosidase staining solution was prepared in accordance with the manufacturer’s instructions. The cells in each well were stained with 1 mL of staining solution after washing twice with PBS. Staining was performed at 37 °C in a dry incubator for 18 h. Any β-Galactosidase-positive cells were considered senescent cells, and at least 200 cells were counted.

### 2.5. Quantitative PCR Analysis

Total RNA was extracted from cultured cells using Sepasol-RNA I Super G (Nacalai Tesque, Kyoto, Japan), following the manufacturer’s protocol. First-strand cDNA synthesis was performed using SuperScript II reverse transcriptase (Invitrogen, MA, USA), with random primers. Quantitative PCR was performed in the presence of KAPA SYBR FAST Universal 2X qPCR Master Mix (Nippon Genetics, Tokyo, Japan), on a Thermal Cycler Dice Real-Time System III (Takara Bio, Kusatsu, Japan), under the following conditions: denaturation at 95 °C for 3 min, followed by 40 cycles at 95 °C for 3 s and 60 °C for 20 s. The primer sets used in this study were as follows: *p16^INK4a^* Fw, ACC AGA GGC AGT AAC CAT GC; *p16^INK4a^* Rv, GGA CCT TCG GTG ACT GAT GA; *GAPDH* Fw, GAA GGT GAA GGT CGG AGT CAA C; *GAPDH* Rv, CAG AGT TAA AAG CAG CCC TGG T. *GAPDH* gene was used as a reference gene for data normalization.

### 2.6. Intracellular Reactive Oxygen Species Scavenging Activity Assay

Cells were seeded in triplicate in 24-well plates and pretreated with HPE for seven days. The cells were then treated with 50 μM H_2_O_2_ for 2 h, followed by the addition of CM-H2DCFDA (Invitrogen, MA, USA), as a general oxidative stress indicator. The cells were stained with Hoechst 33,342 solution (DOJINDO, Tokyo, Japan). After 60 min of treatment with the ROS indicator, images were captured using an all-in-one microscope (BZ-X700, KEYENCE, Osaka, Japan) and analyzed using a BZ-X Analyzer and BZ-X Image Converter (KEYENCE, Osaka, Japan). ROS levels were normalized using Hoechst 33,342 staining.

### 2.7. RNA-Sequencing

Total RNA was extracted from six samples (three biological replicates, each of 50 μM H_2_O_2_-treated NHDFs, and 50 mg/mL HPE + 50 μM H_2_O_2_-treated NHDFs) using the RNAeasy Plus Mini Kit (Qiagen, Venlo, The Netherlands), in accordance with the manufacturer’s instructions. The quality and quantity of the extracted RNA were determined using the Qubit^®^ RNA BR Assay Kit (Invitrogen, Waltham, MA, USA). The libraries were prepared using AmpliSeq cDNA Synthesis for Illumina Kit (Illumina, San Diego, CA, USA), AmpliSeq for Illumina Transcriptome Human Gene Expression Panel (Illumina, CA, USA), AmpliSeq Library PLUS for Illumina (Illumina, CA, USA), and AmpliSeq CD Indexes for Illumina (Illumina, CA, USA), in accordance with the manufacturer’s instructions. The quality and quantity of the libraries were checked using an Agilent 2100 Bioanalyzer (Agilent Technologies, Santa Clara, CA, USA). The libraries were diluted, pooled in accordance with the manufacturer’s recommendations, and sequenced using the MiSeq^®^ system (Illumina, CA, USA). RNA-seq data were deposited in the DNA Data Bank of Japan (DDBJ), Sequence Read Archive (DRA), with accession number DRA014553.

### 2.8. Western Blot Analysis

NHDFs were treated with 10 μM MG132 for 15 h, and total proteins from NHDFs were extracted using an SDS sample buffer. The protein concentration was measured using a BCA protein assay kit. Equal amounts of protein (10 μg) were separated by SDS-PAGE using 4–20% Mini-PROTEAN TGX Gels (Cat# 4561096, Bio-Rad, Hercules, CA, USA) and then transferred onto PVDF membranes. Membranes were blocked using 3% skim milk in Tris-buffered saline, containing 0.1% (*v/v*) Tween-20 (TBST), for 1 h, and incubated with primary antibodies against NRF2 (1:500, Cat#12721, Cell Signaling Technology) and GAPDH (1:2000) overnight at 4 °C, followed by incubation with anti-mouse IgG or anti-rabbit IgG conjugated with horseradish peroxidase. GAPDH was used as an internal control. The probed protein was visualized using ChemiDoc XRS Plus Image Lab (Cat#1708265J1NPC, BIO-RAD). The densitometric analysis was semi-quantified using the Image Lab software (Bio-Rad).

### 2.9. Statistical Analysis

Values are reported as mean ± SEM. Statistical differences were determined using Student’s *t*-test. Statistical significance is indicated as * *p* < 0.05, ** *p* < 0.01, or *** *p* < 0.001.

## 3. Results

### 3.1. HPE Treatment Delays the Onset of Cellular Senescence Induced by Chronic Oxidative Stress

**Figure 1:**
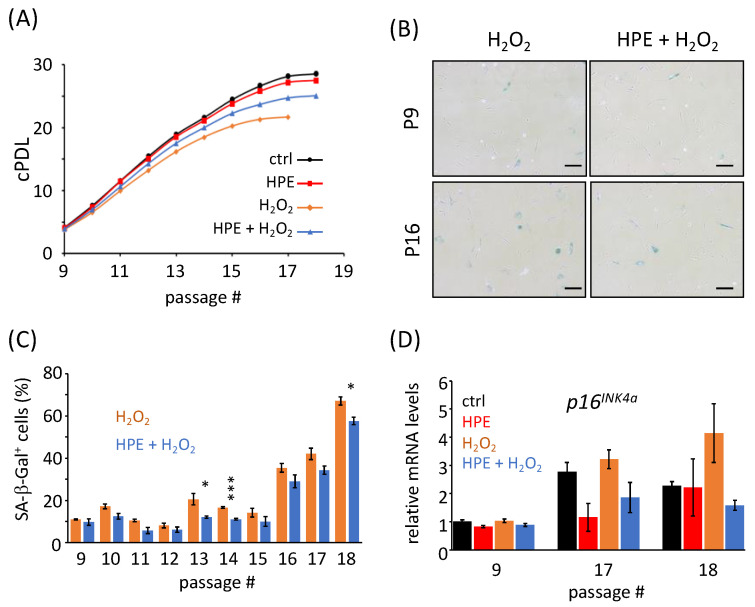
HPE treatment of NHDF cells delays the cellular senescence process. (**A**) Cumulative population doubling level (cPDL) of normal human dermal fibroblasts (NHDFs) with human placental extract (HPE) and/or H_2_O_2_ was measured under indicated conditions. Two independent proliferation assays were performed. (**B**) Pictures of senescence-associated β-Galactosidase (SA-β-Gal) -positive cells (blue) at passages 9 (P9) and 16 (P16), under indicated conditions, were taken. Scale bars represent 100 μm. (**C**) The percentages of SA-β-Gal-positive cells, under indicated conditions, were quantified at different passages. (**D**) *p16^INK4a^* gene expression at different passages, at indicated conditions, was analyzed using qPCR. The expression level under the control (ctrl) condition at P9 was set to 1. * *p* < 0.05, *** *p* < 0.001 was compared between H_2_O_2_ with HPE, and H_2_O_2_ without HPE, within the same passages, by Student’s two-tailed *t*-test.

To investigate whether HPE affects the proliferation of NHDFs under chronic oxidative stress conditions, NHDFs were cultured until they ceased to proliferate, under H_2_O_2_ treatment, with or without 500 μg/mL HPE, as used in our previous study [23]. We preliminarily treated NHDFs with H_2_O_2_ at 50, 100, 200, or 300 μM. Treatment with H_2_O_2_ at concentrations greater than 100 mM was too severe to proliferate; however, treatment with 50 μM H_2_O_2_ demonstrated a modest effect on proliferation. Therefore, we decided to treat NHDFs at 50 μM for subsequent experiments in this study. Cessation of cell proliferation (CCP) was defined by an iPDL value of < 0.5. Untreated and H_2_O_2_-treated NHDFs reached CCP at passages 18 and 17, with a cPDL of 28.58 ± 0.43 and 21.65 ± 0.28, respectively (Figure 1A). These values were significantly lower than those of the control cells (*p* = 2.7 × 10^−4^), suggesting the early onset of senescence, with lower cPDL compared to that in the control cells. Notably, NHDFs treated with both H_2_O_2_ and HPE reached CCP at passage 18, with a cPDL of 25.07 ± 0.43 (Figure 1A), which was significantly higher than that of H_2_O_2_-treated NHDFs (*p* = 3.4 × 10^−3^). Notably, CCP and cPDL of NHDFs treated with HPE alone were comparable to those of the control NHDFs (CCP, 18; cPDL, 27.51 ± 0.43; *p* = 0.21). These results indicate that HPE treatment enhances cPDL under chronic oxidative stress conditions, but not under normal conditions.

To confirm whether HPE treatment attenuates chronic oxidative stress-induced cellular senescence, we performed a senescence-associated β-galactosidase (SA-β-Gal) assay and determined the gene expression levels of *p16^INK4a^*, a senescent marker. Under all conditions, the ratios of SA-β-Gal-positive cells increased with passages (Figure 1B,C and Supplementary Figure S1). In addition, H_2_O_2_-treated NHDFs possessed a higher number of SA-β-Gal-positive cells than the control cells within the same passages (Supplementary Figure S1A). No differences were observed in the ratio of SA-β-Gal-positive cells between the control and HPE-treated NHDFs within the same passages (Supplementary Figure S1B), which is consistent with the proliferation assay results (Figure 1A). In contrast, the HPE + H_2_O_2_-treated NHDFs possessed fewer SA-β-Gal-positive cells than the H_2_O_2_-treated NHDFs within all passages (Figure 1B,C). Consistent with the results of the SA-β-Gal assay, the HPE + H_2_O_2_-treated NHDFs expressed less *p16^INK4a^* mRNA than H_2_O_2_-treated NHDFs at higher passages (Figure 1D). Collectively, these results demonstrate that HPE treatment attenuates chronic oxidative stress-induced cellular senescence.

### 3.2. HPE Enhances the Scavenging of Cellular ROS Levels

**Figure 2:**
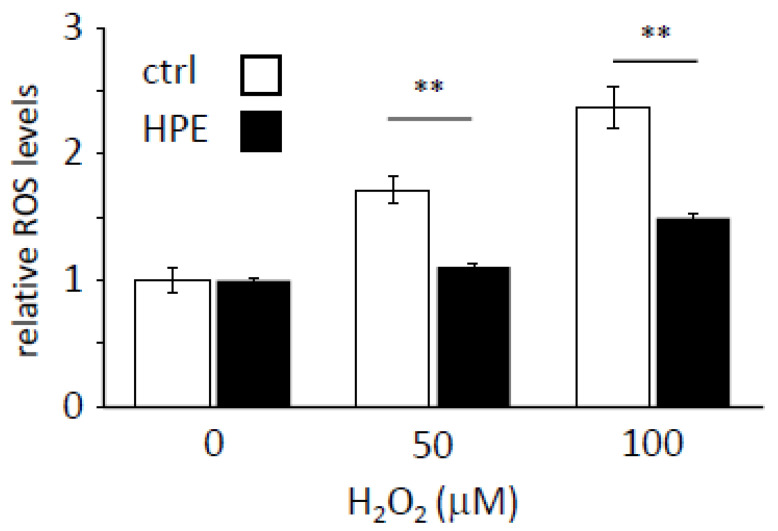
HPE treatment increases the ROS scavenging potential. Cellular reactive oxygen species (ROS) levels were measured under the indicated H_2_O_2_-treated conditions with or without human placental extract (HPE). The ROS level under the control condition, without HPE, was set to 1. Sample numbers were 3–4 in each condition. ** *p* < 0.01 by Student’s two-tailed *t*-test.

Placental extracts have shown antioxidant properties in both in vitro and in vivo models [17,18,19,20,21,22]. Therefore, we investigated whether HPE enhances ROS scavenging in H_2_O_2_-treated NHDFs. ROS levels increased in an H_2_O_2_ dose-dependent manner in cells with and without HPE pretreatment, but HPE-treated cells had lower ROS levels than non-treated cells, at each H_2_O_2_ concentration (Figure 2). This result indicates that HPE could activate the ROS-scavenging pathway in NHDFs.

### 3.3. HPE Upregulates a Set of Antioxidant Genes under H_2_O_2_-Treated Conditions

**Figure 3:**
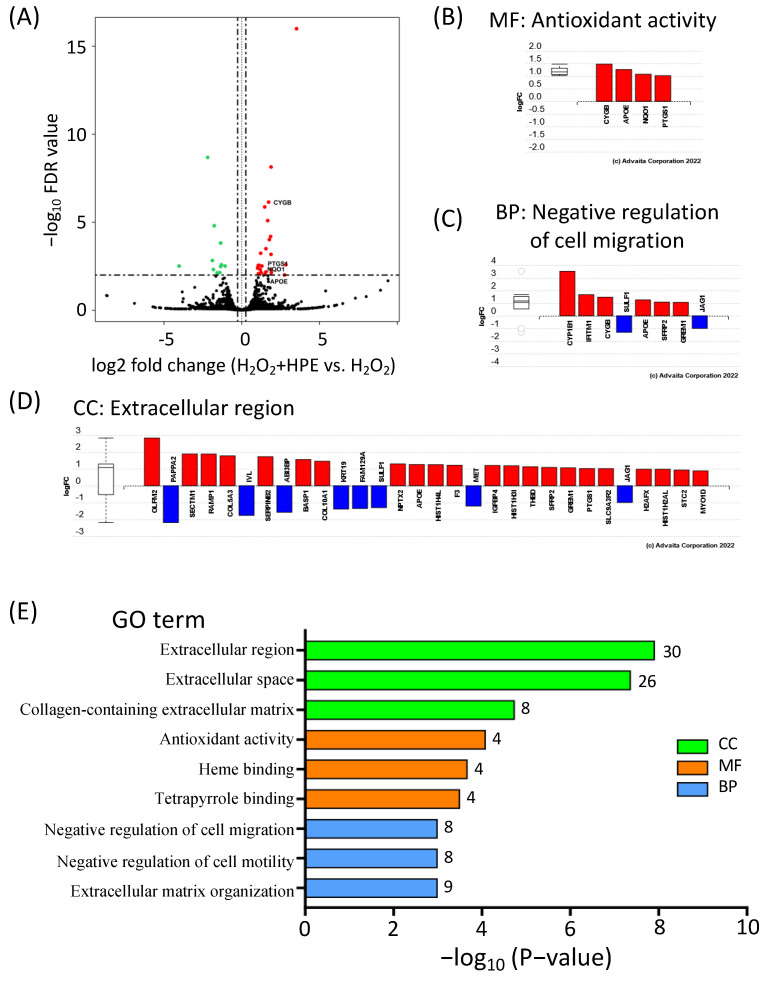
HPE increases antioxidant gene expression levels in H_2_O_2_-treated NHDFs. (**A**) Volcano plot of differentially expressed genes (DEGs) of H_2_O_2_-treated NHDFs with HPE. Red, black, and green dots represent upregulated, non-differentially expressed, and down-regulated genes, respectively. (**B**–**D**) DEGs under the top-identified molecular function (MF), biological process (BP), and cellular components (CC). Red and blue bars represent up-, and downregulated genes, respectively. (**E**) The top 3 significantly enriched GO terms in the CC (green), MF (orange), and BP (blue).

The increase in ROS scavenging in HPE-treated NHDFs prompted us to investigate which antioxidant genes were upregulated by HPE treatment under oxidative stress conditions. To address this, we performed RNA-sequencing using H_2_O_2_-treated NHDFs, with or without HPE treatment. The significantly differentially expressed genes (DEGs) of the HPE + H_2_O_2_-treated NHDFs were defined by a false discovery rate (FDR) with a *p*-value of <0.05 and a fold change (FC) of >1.2. The gene expression profiles of the pretreated cells were analyzed and the expression patterns were represented by a volcano plot (Figure 3A), where the green dots represent the significantly downregulated DEGs, and the red dots represent the significantly upregulated genes. The results revealed that among the 62 DEGs, 43 were upregulated and 19 were downregulated.

In the HPE + H_2_O_2_-treated NHDFs, gene ontology (GO) enrichment analyses categorized the 62 DEGs into three major categories: biological process (BP), molecular function (MF), and cellular component (CC) (Figure 3B–E and Supplementary Table S1). Notably, “antioxidant activity” received the highest score in the MF category, in which all four DEGs, *CYGB*, *APOE*, *NQO1*, and *PTGS1*, were upregulated (Figure 3B). The top two GO terms in the MF were “heme binding” and “tetrapyrrole binding”. The three most significant BP GO terms were “negative regulation of cell migration”, “negative regulation of cell motility”, and “extracellular matrix organization” (Figure 3C,E, and Supplementary Dataset S1). For the CC category, the three most significant GO terms were “extracellular region”, “extracellular space”, and “collagen-containing extracellular matrix” (Figure 3D,E, and Supplementary Dataset S1). Among these DEGs, many were found to be related to the extracellular matrix, which is consistent with our previous study [23]. Collectively, GO enrichment analyses revealed that HPE treatment upregulated a set of antioxidant genes, suggesting that HPE treatment has the potential to protect cutaneous tissues against oxidative stress caused by photoaging in vivo.

### 3.4. HPE Increases NRF2 Protein Levels under Oxidative Stress

**Figure 4:**
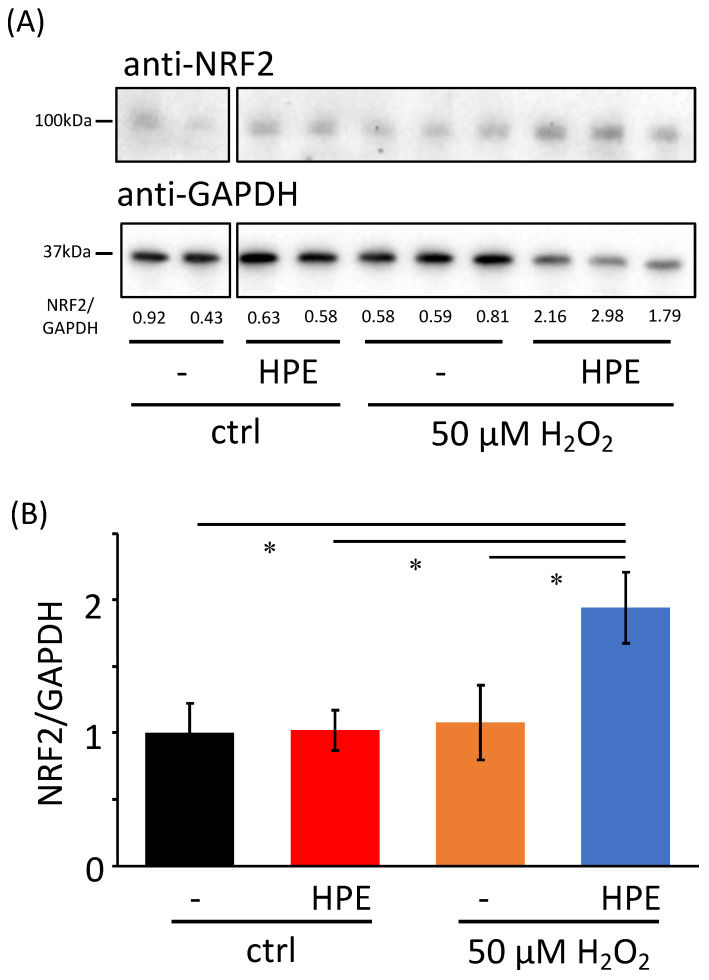
Increased NRF2 protein levels in H_2_O_2_-treated NHDFs with HPE. (**A**) NRF2 (upper panel) and GAPDH (bottom panel) protein levels, under indicated conditions, were detected. Numbers are relative NRF2 intensities, normalized by GAPDH, quantified using Image Lab software. (**B**) The bar graph shows a relative densitometry representation of (**A**). Sample numbers were 7 in each condition. Paired *t*-test; * *p* < 0.05 by Student’s two-tailed *t*-test.

Finally, we investigated the mechanism by which HPE attenuates oxidative stress in NHDFs. Since the NRF2/KEAP1 pathway is the major regulator of cytoprotective responses to oxidative stress [7], we first confirmed whether *NFE2L2* and *KEAP1* gene levels, which encode *NRF2* and *KEAP1* mRNAs, respectively, were altered by HPE treatment. However, there were no differences between H_2_O_2_-treated cells, with or without HPE (*NFE2L2*: LogFC −0.650, *p* = 0.833, *KEAP1*: LogFC 0.200, *p* = 0.956). In contrast, we observed that total NRF2 protein levels increased approximately two-fold in HPE + H_2_O_2_-treated cells (Figure 4). Furthermore, we showed that HPE or H_2_O_2_ treatment alone did not affect NRF2 protein levels. These results imply that HPE post-transcriptionally, and/or post-translationally, upregulates NRF2 protein levels under oxidative stress conditions, which may enable NHDFs to alleviate oxidative stress.

## 4. Discussion

Chronic sunlight exposure, especially from UVR, is the most harmful environmental factor for cutaneous health and triggers photoaging. UVR causes DNA damage, such as strand breaks, crosslinks, and base modifications [25], as well as oxidative stress, both of which contribute to the accumulation of senescent keratinocytes and/or fibroblasts in cutaneous tissue. In this study, we revealed that HPE delays the in vitro cellular senescence process, via accelerated chronic H_2_O_2_ treatment, mimicking chronic UVR exposure of cutaneous tissue in NHDFs. Our findings suggest that chronic subcutaneous injection of HPE in humans may impair photoaging.

NRF2 transcriptional activity is determined by its subcellular localization, and nuclear NRF2 is the active form. In this study, we did not analyze NRF2 subcellular localization; however, we observed that NRF2 protein levels increased after H_2_O_2_ and HPE treatment. This result implies that NRF2 transcriptional activity is higher under H_2_O_2_ + HPE-treated conditions, which likely protects NHDFs from oxidative stress and delays the onset of cellular senescence. Based on our GO analysis, HPE treatment under oxidative stress enhanced the expression of the antioxidant genes *CYGB*, *APOE*, *NQO1*, and *PTGS1*. Among these, *CYGB*, *APOE*, and *NQO1* are reportedly upregulated by NRF2 [26,27], supporting our interpretation that HPE treatment enhances NRF2 transcriptional activity. In contrast, typical NRF2-dependent antioxidant genes, such as *SOD2* (logFC −0.159; *p* = 0.833) and *CAT* (logFC 0.627; *p* = 0.542), were not upregulated, even under these conditions, suggesting that further investigations are required in order to elucidate the overall molecular mechanisms of the HPE-mediated antioxidant pathway.

As mentioned earlier, NRF2 protein levels increased in H_2_O_2_ + HPE-treated cells. However, DEGs analysis revealed that *NRF2* mRNA expression level, which is encoded in humans by the *NFE2L2* gene, under H_2_O_2_ + HPE-treated conditions was comparable to that under H_2_O_2_-treated conditions (logFC −0.650; *p* = 0.833). This raises two possibilities. The first possibility is a change in the NRF2 protein stability. NRF2 protein levels are tightly regulated by the KEAP1-dependent ubiquitin-proteasome system to maintain low levels under normal conditions [28,29]. Upon exposure to electrophilic/oxidative stress, NRF2 becomes free from KEAP1, whereafter it stabilizes and translocates into the nucleus, to activate the transcription of a series of antioxidant genes [7]. Therefore, HPE treatment under oxidative conditions may post-translationally modify KEAP1 and/or NRF2 to reduce the NRF2/KEAP1 affinity. Another possibility is miRNA-mediated post-transcriptional regulation of *NRF2* mRNA. Several miRNAs reportedly suppress the translation of *NRF2* mRNA [30]. Therefore, HPE treatment under oxidative stress conditions might downregulate miRNA expression and induce the translation of *NRF2* mRNA. The molecular mechanisms of HPE-mediated NRF2 regulation are largely unknown but are of substantial interest.

The HPE used in this study contains a wide array of amino acids, peptides, nucleic acids, and lipids [10,24] and is approved for subcutaneous injection as a prescription drug for the treatment of menopausal disorders. Although we have not identified the factors responsible for regulating NRF2 and/or antioxidant gene expression, investigations to identify these factors will be critical for revealing the molecular mechanisms of the HPE-mediated delay of cellular senescence. Furthermore, examining whether HPE treatment reduces senescent cells in vivo and benefits skin health in animals and humans would be crucial.

## 5. Conclusions

**Figure 5:**
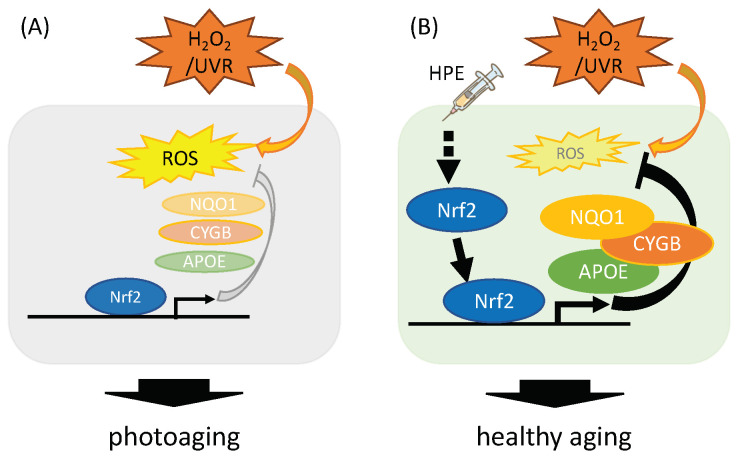
Schematic representation of the HPE-dependent regulation on antioxidant machinery. (**A**) Under normal conditions, H_2_O_2_/UVR produces ROS, which promotes cellular senescence and photoaging. (**B**) Under HPE-treated conditions, HPE increases the active form of NRF2, via unknown mechanisms, and decreases cell susceptibility to oxidative stress, thereby delaying cellular senescence and possibly improving skin aging.

In this study, we demonstrated, for the first time, that HPE delayed cellular senescence in vitro. We further revealed that, under oxidative stress conditions, HPE increased NRF2, a vital molecule in the antioxidant pathway; it also upregulated antioxidant gene expression, and accelerated ROS scavenging. These findings imply that chronic HPE-treated NHDFs, under oxidative stress conditions, delay cellular senescence by mitigating oxidative stress via upregulation of the NRF2-mediated antioxidant pathway (Figure 5), suggesting that chronic HPE treatment might ameliorate skin-aging-associated damage in vivo. These findings are consistent with previous clinical evidence that HPE improves skin health.

## Data Availability

The dataset generated in this study is available in the DNA Data Bank of Japan (DDBJ) Sequence Read Archive (DRA) with the accession number DRA014553.

## Supplementary Materials

The following supporting information can be downloaded at: https://www.mdpi.com/article/10.3390/antiox11081545/s1, Figure S1: Serially passaging of NHDFs increased SA-β-Gal-positive cells in all conditions, Table S1: Top identified GO terms for three categories: biological processes, molecular functions, and cellular components; Dataset S1: DEGs in the top identified GO terms for the three categories.
