# Supplementary material for: Human Placental Extract Delays In Vitro Cellular Senescence through the Activation of NRF2-Mediated Antioxidant Pathway
Source: Antioxidants (Basel). 2022 Aug 10;11(8):1545. doi: 10.3390/antiox11081545 (PMC9405396; doi:10.3390/antiox11081545)
Supplement: Supplementary file 1 [file antioxidants-11-01545-s001.zip › Figure S1.pptx]

## Slide 1
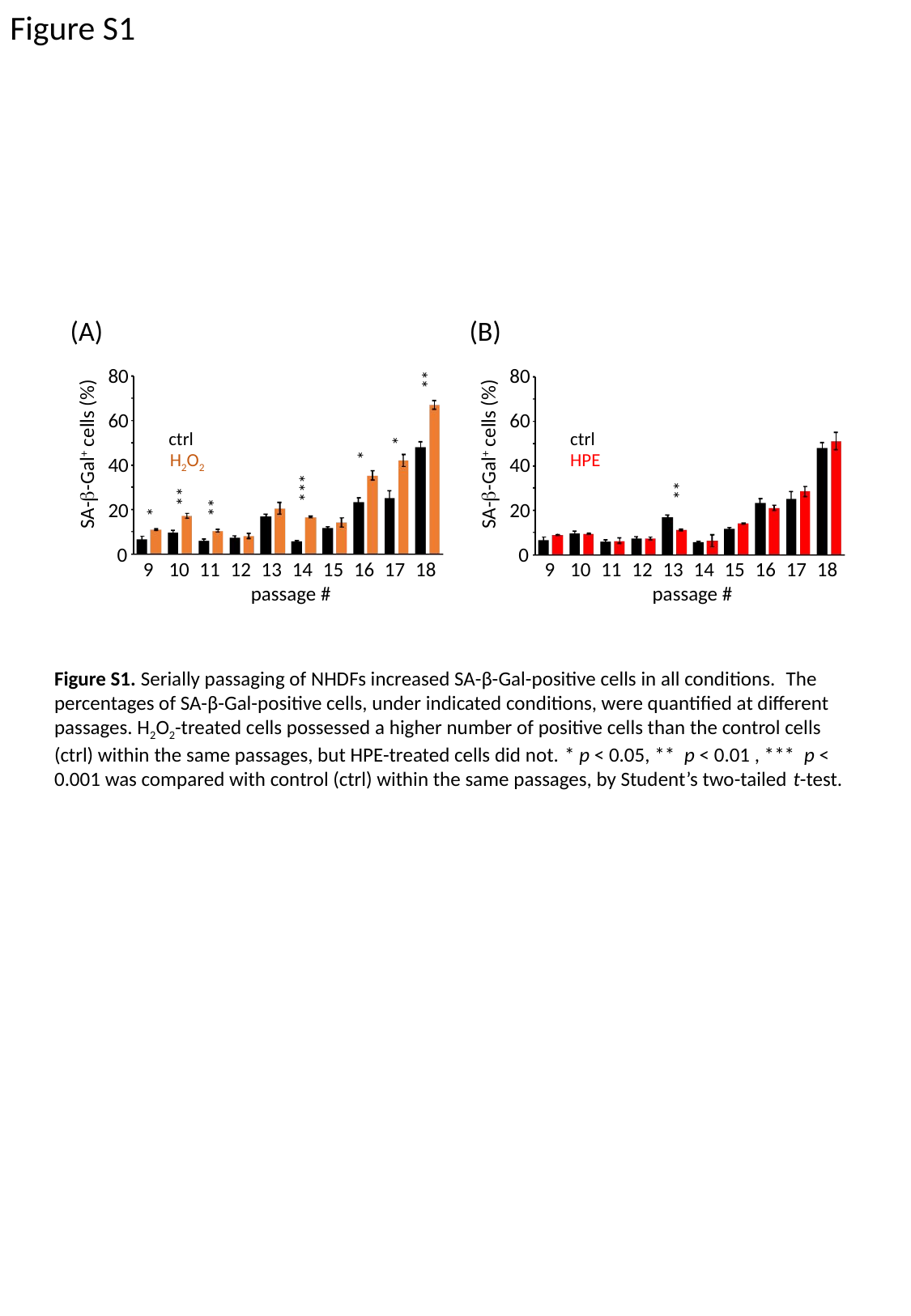

Figure S1
(A)
(B)
80
60
SA-b-Gal+ cells (%)
40
20
0
9
10
11
12
13
14
15
16
17
18
passage #
**
ctrl
*
*
H2O2
***
**
**
*
80
60
SA-b-Gal+ cells (%)
40
20
0
9
10
11
12
13
14
15
16
17
18
passage #
ctrl
HPE
**
Figure S1. Serially passaging of NHDFs increased SA-β-Gal-positive cells in all conditions.  The percentages of SA-β-Gal-positive cells, under indicated conditions, were quantified at different passages. H2O2-treated cells possessed a higher number of positive cells than the control cells (ctrl) within the same passages, but HPE-treated cells did not. * p < 0.05, ** p < 0.01 , *** p < 0.001 was compared with control (ctrl) within the same passages, by Student’s two-tailed t-test.
