# Supplementary material for: Human Placental Extract Delays In Vitro Cellular Senescence through the Activation of NRF2-Mediated Antioxidant Pathway
Source: Antioxidants (Basel). 2022 Aug 10;11(8):1545. doi: 10.3390/antiox11081545 (PMC9405396; doi:10.3390/antiox11081545)
Supplement: Supplementary file 1 [file antioxidants-11-01545-s001.zip › Supplementary Table S1.pptx]

## Slide 1
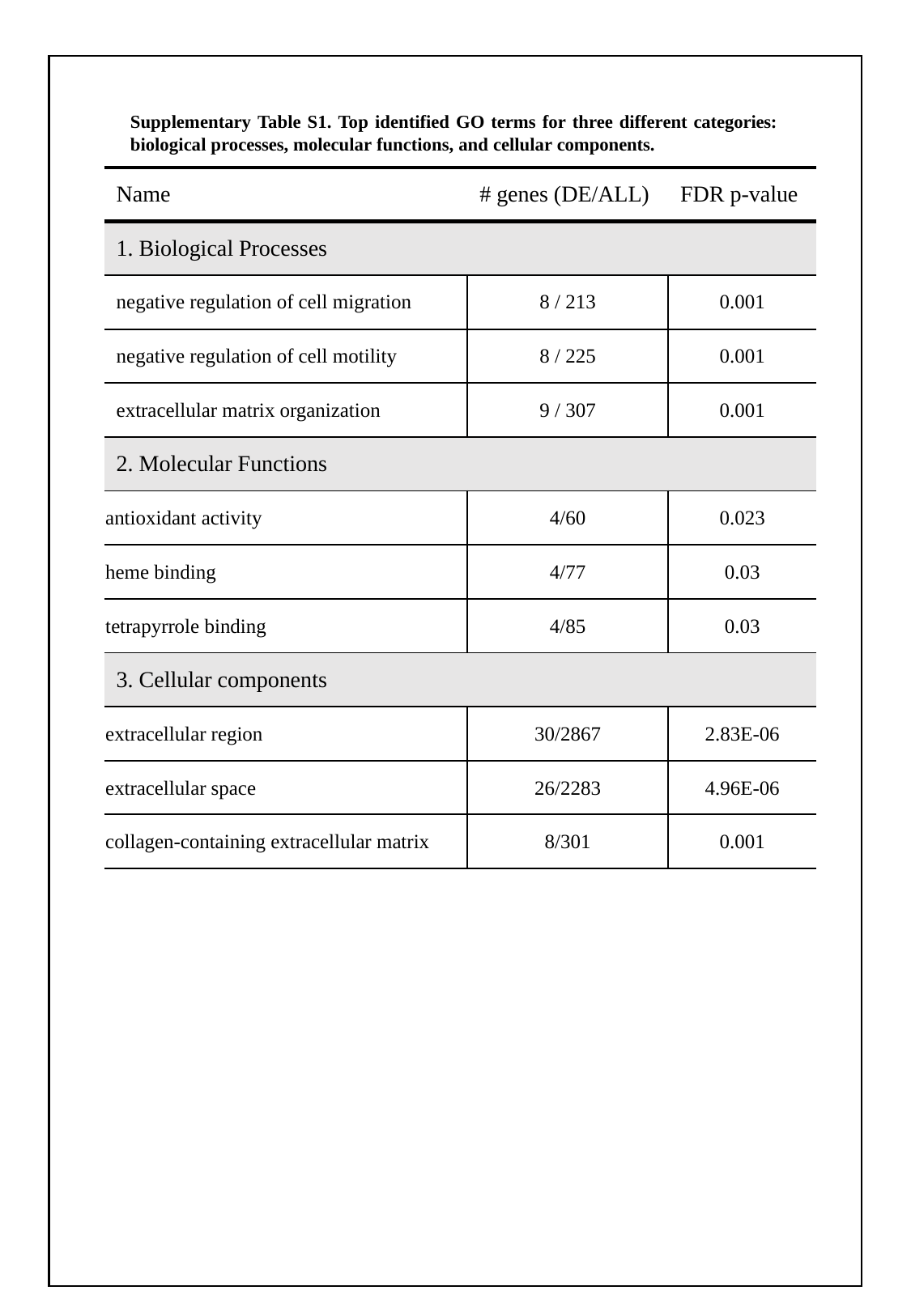

Supplementary Table S1. Top identified GO terms for three different categories: biological processes, molecular functions, and cellular components.
| Name | # genes (DE/ALL) | FDR p-value |
| --- | --- | --- |
| 1. Biological Processes | | |
| negative regulation of cell migration | 8 / 213 | 0.001 |
| negative regulation of cell motility | 8 / 225 | 0.001 |
| extracellular matrix organization | 9 / 307 | 0.001 |
| 2. Molecular Functions | | |
| antioxidant activity | 4/60 | 0.023 |
| heme binding | 4/77 | 0.03 |
| tetrapyrrole binding | 4/85 | 0.03 |
| 3. Cellular components | | |
| extracellular region | 30/2867 | 2.83E-06 |
| extracellular space | 26/2283 | 4.96E-06 |
| collagen-containing extracellular matrix | 8/301 | 0.001 |
